# Tonicity of oral rehydration solutions affects water, mineral and acid–base balance in calves with naturally occurring diarrhoea

**DOI:** 10.1111/jpn.13405

**Published:** 2020-07-04

**Authors:** Juliette N. Wilms, Juanita Echeverry‐Munera, Lauren Engelking, Leonel N. Leal, Javier Martín‐Tereso

**Affiliations:** ^1^ Trouw Nutrition R&D Amersfoort The Netherlands; ^2^ Department of Animal Bioscience University of Guelph Guelph ON Canada; ^3^ Agricultural, Food & Nutritional Science University of Alberta Edmonton AB Canada

**Keywords:** acid–base balance, base excess, calf diarrhoea, mineral balance, strong ion difference, tonicity

## Abstract

Recommendations for composition of oral rehydration solutions (ORS) for calves, particularly concerning Na^+^, glucose, and their combined effect on tonicity, are not in line with guidelines for humans. Thus, this study aimed to determine the effect of ORS tonicity on water, mineral and acid–base balance. Seventy‐two calves were selected based on the severity of dehydration and blood base excess (BE) on day 0. Five calves that did not develop diarrhoea were removed post‐inclusion from the study. Calves were allocated to blocks of four animals based on blood BE on day 1. Within each block, calves were randomly assigned to one of four treatments: (a) hypotonic ORS with low Na^+^ and lactose (HYPO); (b) isotonic ORS with low Na^+^and glucose (ISO); (c) hypertonic ORS with high Na^+^ and glucose (HYPER); and (d) control consisting of warm water including 5 g/L of whey powder (CON). Treatments were administered twice daily over a 3‐day period, in which calves were offered 2.0 L of treatment at 1300 and 2100 hr. Calves were fed 2.5 L of milk replacer at 0700 and 1630 hr from day 1 to 3 and 3.0 L from day 4 to 5, and had access to water. Calves were monitored for 5 days in which measurements included intakes, BW, blood sampling and collection of faeces on day 1 and urine from day 1 to 3. All ORS treatments maintained normal serum Na^+^, whereas CON did not. Calves in the HYPER group had lower blood pH and greater faecal Na^+^ losses than HYPO and ISO. Plasma expansion relative to baseline was higher in low tonicity ORS (+4.8%) when compared with CON (+1.0%). Urine osmolality was 30% higher in HYPER calves. In this experiment, low tonicity ORS were more effective at restoring water, mineral and acid–base balance than the hypertonic ORS.

## 1 INTRODUCTION

A major objective of diarrhoea treatment in calves is to mitigate the severity of dehydration and metabolic acidosis, which involves administration of oral rehydration solutions (ORS; Constable, Thomas, & Boisrame, 2001). Oral rehydration solutions were originally developed in human medicine during the 1960s for rehydration of patients with cholera infections. Although much research has been done on oral rehydration therapy, considerable variability exists in the composition, and thus effectiveness, of commercially available ORS for calves. Recommendations for the appropriate ORS, particularly regarding sodium (Na^+^) and glucose concentrations, and therefore tonicity, are conflicting between human and veterinary medicine (Michell, 2005). The World Health Organization (WHO) recommends an ORS with an osmolality of 245 mOsm/kg, including 75 mM of Na^+^ and 75 mM of glucose, regardless of the aetiology and the severity of the diarrhoea (UNICEF & WHO, 2016). In patients with cholera, hypotonic ORS reduces stool output, vomiting incidence and the need for intravenous fluid infusions compared to isotonic ORS (Bhatnagar, 2001; Santosham et al., 1996).

*Cryptosporidium parvum* and Rotavirus represent approximately half of all diarrhoea cases in calves within the first 3 weeks of life (Bartels, Holzhauer, Jorritsma, Swart, & Lam, 2010; Cho & Yoon, 2014). These pathogens cause malabsorptive diarrhoea, where faecal Na^+^ losses are lower than those of diarrhoea induced by the enterotoxigenic *Escherichia coli K99* and cholera (Molla, Rhman, Sarker, Sack, & Molla, 1981; Raizada et al., 1992; Foster & Smith, 2009). Malabsorptive diarrhoea is caused by a reduction of the absorptive surface often due to intestinal mucosal damages (Klein, Kleinová, Volek, & Šimůnek, 2008). This results in decreased intestinal absorption of carbohydrate, protein, fat, minerals or vitamins. Despite the physiological differences linked with the diarrhoea aetiology, ORS for calves usually contain much higher Na^+^ (90–130 mM) and glucose (100–260 mM) concentrations than the ORS recommended by the WHO (Smith & Berchtold, 2014). As a consequence, higher Na^+^ concentrations, and thus higher tonicity of ORS may exceed the absorptive capacity of diarrhoeic calves.

In diarrhoeic calves, hypertonic ORS have shown to increase extracellular fluid volume (Michell, Brooks, White, & Wagstaff, 1992), to contribute to higher plasma glucose (Constable et al., 2001), to minimize weight loss (Brooks, Utiite, Wagstaff, & Michell, 1996) and to improve survival rates (Naylor, Petrie, Rodriguez, & Skilnick, 1990) when compared with lower tonicity ORS. However, in all previously mentioned experiments, milk was either withheld from 24 to 48 hr or the alkalinizing capacity of the tested ORS was not balanced across treatments, thus making efficacy comparison between low and high tonicity ORS difficult. Literature shows that ORS should be fed alongside regular milk provision, as withholding milk or feeding lower milk levels exacerbates weight loss, dehydration and prolongs recovery from diarrhoea (Garthwaite, Drackley, McCoy, & Jaster, 1994; Ollivett, Nydam, Linden, Bowman, & Van Amburgh, 2012). Other authors successfully tested hypertonic ORS (Na^+^> 130 mM) administered in between milk meals on diarrhoeic calves, but without including reference groups with lower tonicity ORS (Sayers, Kennedy, Krump, Sayers, & Kennedy, 2016; Stampfli, Oliver, & Pringle, 2012).

The most concerning health issue related to hypertonic ORS is hypernatremia, where clinical signs include digestive tract upsets, central nervous system dysfunction and death in acute cases (Pringle & Berthiaume, 1988; Wilms, Leal, & Martín‐Tereso, 2020). The excess of solutes present in hypertonic solutions may also worsen the diarrhoea severity by further stimulating water efflux to the lumen of the gut (Lifshitz & Wapnir, 1985). Furthermore, ORS with high osmolality (>650 mOsm/kg) have been shown to significantly impair abomasal emptying rates (Hildebrandt et al., 2020; Sen, Constable, & Marshall, 2006), potentially increasing the incidence of abomasal bloat in calves (Burgstaller, Wittek, & Smith, 2017). Low tonicity ORS may therefore be safer and more effective in diarrhoeic calves continuing to receive whole milk or milk replacer (MR). Thus, this study aimed to determine the effect of ORS tonicity (as driven by NaCl, glucose and lactose) on water, mineral and acid–base balance in calves fed MR and with naturally occurring diarrhoea. Additionally, the effect of replacing glucose by lactose was investigated, as the use of lactose allows a partial reduction of ORS tonicity.

## 2 MATERIALS AND METHODS

This study was conducted at the Calf Research Facility of Trouw Nutrition Research and Development (Sint Anthonis, the Netherlands) between July and August 2017.

### 2.1 Animals and Experimental Design

Holstein‐Friesian male calves (22 ± 7 days of age) with naturally occurring diarrhoea were enrolled in a complete randomized block design. Calves were acquired on day 0 from a collection centre with a capacity of 1,300 calves (Ibbenbüren, Germany). Animals with low BW (<55 kg) were the target animals as low BW in collection centres has been associated with higher morbidity and mortality in veal facilities by Renaud et al. (2018). Animals presenting signs of dehydration, such as a higher degree of enophthalmos and a delayed skin turgor, were grouped in one pen of approximately 20 calves. Blood was then collected from the jugular vein to determine blood base excess (BE) using a portable blood gas analyser (VetScan I‐STAT®1, ref: 600–7015), and consequently, calves with the lowest blood BE and that did not present other health issues than diarrhoea were included in the study. Calves brought into the facility in the same week were considered one batch (*n* = 12). Between July and August 2017, six batches of calves were acquired for this study for a total of 72 calves staggered over the duration of the experiment. On the day after arrival (day 1), a second blood sample was taken, and calves were allocated to blocks of four animals based on blood BE values. Within a block, calves were randomly assigned to one of four treatments including (a) hypotonic ORS with low Na^+^ and low lactose (HYPO, *n* = 18); (b) isotonic ORS with low Na^+^ and low glucose (ISO, *n* = 18); (c) hypertonic ORS with high Na^+^ and high glucose (HYPER, *n* = 18); and (d) a control solution consisting of 5 g/L of whey powder (CON, *n* = 18). All ORS treatments used in this experiment were experimental formulas manufactured by Trouw Nutrition (Putten, the Netherlands) and which did not include any additional components than those described in Table 1. Each of the three ORS were formulated to have the same alkalinizing capacity (strong ion difference [SID] of 75 mEq/L, and propionate concentration of 72 mM) and a glucose to sodium ratio of 1.13. The concentration of the CON treatment was designed to have no effect on the health of the animals. Allocation to treatment was performed by a person who was not involved in treatment administration and sampling. Treatments were blinded to animal caretakers by randomly assigning a letter (A, B, C or D) to each treatment. Treatments were administered daily, from day 1 to 3, during which calves were offered 2.0 L of treatment at 1300 and 2100 hr. Treatments were reconstituted with water and supplied in a teat bucket at 40°C. Calves were allowed to consume the solutions for 3 hr starting at the time of supply. Treatments were never drenched to determine voluntary consumption. Milk replacer formula consisted of 50.0% skimmed milk powder, 19.5% whey powder, 24.2% of a spray‐dried fat concentrate, 4.8% of whey protein concentrates (Trouw Nutrition, Deventer, The Netherlands) and 1.5% of mineral, amino acids and vitamin MR supplements (Trouw Nutrition, Putten, The Netherlands). This resulted in 41.5% lactose, 25.0% crude oils and fats, 22.5% crude protein and 6.8% ash on DM basis. Milk replacer was reconstituted with water at a concentration of 150 g/L (15% solids, 389 mOsm/kg) and supplied in a teat bucket at 40°C. Milk replacer was formulated to have a similar Na^+^ concentration and SID as bovine whole milk to avoid interference with the ORS treatments, especially concerning the alkalinizing capacity of the products. Osmolality of the MR was higher than that of bovine whole milk (~300 mOsm/kg), which is common in MR for calves due to higher levels of lactose and ash (Wilms, Berends, & Martín‐Tereso, 2019; Wilms, Berends, Leal, & Martín‐Tereso, 2020). When calves arrived at the research facility on the evening of day 0, they were offered 2.5 L of MR. Calves were then fed 2.5 L of MR from day 1 to 3, and 3.0 L on day 4 and 5, twice daily at 0630 and 1700 hr. Calves were allowed to consume MR for 15 min starting at the time of supply. Calves from all treatment groups were provided with ad libitum access to plain water through a bucket except for the 6‐hr duration of treatment supply to facilitate intake of treatments. No solid feed was provided during the five monitoring and sampling days. Animals displaying severe signs of dehydration (>8% BW; Smith & Berchtold, 2014) and metabolic acidosis (BE < −15 mM) were excluded from the study and provided with an intravenous saline and glucose infusion, as well as additional veterinary care. Excluded calves were not replaced by new animals.

**TABLE 1:** Descriptive summary of milk replacer and treatment components fed to calves with naturally occurring diarrhoea receiving either a control solution or oral rehydration solutions (ORS) with various tonicities (*n* = 66)

Item^1^	Milk replacer^2^	Treatments^3^				Conventions for calf ORS^4^
		CON	HYPO	ISO	HYPER	
Sugars						
Lactose	190	10	45	0	0	–
Glucose	25	0	0	90	151	100–260
Minerals						
Sodium	32	2	80	80	134	90–130
Potassium	48	3	27	27	27	10–30
Chloride	41	2	33	33	86	40–60
Calcium	33	1	0	0	0	–
Phosphate	41	0	0	0	0	–
Magnesium	8	0	0	0	0	–
Alkalinizing agents						≥50
Propionate	0	0	72	72	72	–
SID (mEq/L)^5^	39	3	74	74	75	≥60
Glu:Na	–	12	1.13	1.13	1.13	1.1–3.1
Osmolality (mOsm/kg)	389	18	257	302	470	400–600

^1^ Expressed in mM unless specified otherwise.

^2^ Commercial milk replacers for calves often contain higher levels of minerals (ash fraction) and lactose than bovine whole milk. Consequently, the osmolality of MR can range from slightly hypertonic (just above 300 mOsm/kg) to highly hypertonic (>450 mOsm/kg; Wilms et al., 2019; Wilms, Berends, et al., 2020).

^3^ Treatments included three ORS: low Na^+^, low lactose (HYPO, *n* = 16), low Na^+^, low glucose (ISO, *n* = 16), high Na^+^, high glucose (HYPER, *n* = 16), and one control solution consisting of 5 g/L of whey powder (CON, *n* = 18).

^4^ Recommendation for ORS formulation for calves (Smith & Berchtold, 2014).

^5^ Effective strong ion difference (SID) = (Na^+^ + K^+^) − Cl^−^.

### 2.2 Housing

Calves were housed indoors in individual pens (1.22 × 2.13 m), separated by plastic solid dividers, with 50% laying area covered with flax straw in the back. During total faecal and urine collection, calves were tethered to the front of the pen and an elevated plateau covered with rubber was added to the front of the pen to elevate the animals to ease urine collection. The temperature in the calf facility was maintained at a minimum of 15°C and a maximum of 28°C and relative humidity between 60% and 85%. Calves were exposed to daylight and artificial light from 0600 to 2200 hr, and a nightlight for the remainder of the day.

### 2.3 Measurements

**FIGURE 1:**
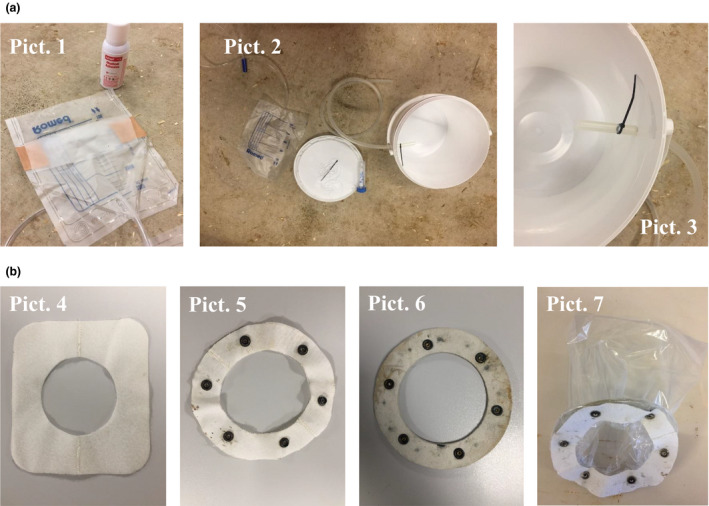
Devices used for total urine collection (a) and total faecal collection (b). In Figure a, picture 1 displays how urine catheter bags for human are transformed to urine collection bags that are sealed on the sides to allow urine to flow into the tube. Medical glue was used to glue both sides of the bag to the calf. Pictures 2 and 3 display how the tube from the collection bag is connected to the bucket, which was placed at a lower level than the calf to allow urine to flow into the bucket. In Figure b, picture 4 displays the Velcro material that was glued to the hindquarter of the calf. Pictures 5 and 6 show the rings, one Velcro, one leather, that were used to attach the faecal collection bag (Picture 7) to the Velcro part shown in picture 4. The plastic bag containing faeces was changed regularly throughout the collection period. Parts glued to the calf remained in place until the completion of the collection period

A representative sample of MR (100 g) and treatment components (100 g) were collected for analysis prior to the start of the study. All intakes were recorded from day 1 to 5. Calf BW was measured on day 1 and 5 between 1100 and 1200 hr. At the collection centre, blood samples were taken in the morning of day 0 into 9‐ml tubes with anticoagulant (lithium heparin, BD Vacutainer, BD, Vianen, the Netherlands) and were analysed immediately using CG8^+^ cartridges for blood pH, BE, Na^+^ and glucose using a portable blood gas analyser (VetScan I‐STAT^®^1, ref: 600–7015). During the five monitoring days, blood samples were collected daily from the jugular vein at 1100 hr. Blood samples were taken in two 9‐ml tubes with anticoagulant (lithium heparin) and two 9‐ml serum tubes (BD Vacutainer, BD, Vianen, the Netherlands). For blood acid–base (pH, BE and HCO_3_), blood gas determination (total carbon dioxide [tCO_2_], partial pressure of carbon dioxide [pCO_2_]) and l‐Lactate measurements, one drop of whole blood from the lithium heparin tube was inserted into CG4^+^ cartridges and analysed immediately using the blood gas analyser mentioned above. Haematocrit (Htc) was determined immediately in whole blood from the lithium heparin tube via capillary centrifugation using a centrifuge (Haematokrit 200, Hettich, Tuttlingen, Germany). Samples were centrifuged at  3,850 *g* for 5 min at ambient temperature. Serum tubes were set for 15 min and centrifuged at 1,500 *g* for 15 min at 20°C (Rotina 380 R, Hettich, Tuttlingen, Germany). Tubes with lithium heparin were centrifuged at 1,500 *g* for 15 min at 4°C. Plasma and serum aliquots were stored in 1.5‐mL cryotubes at −18°C. Faeces were quantitatively collected in the first 24 hr and urine in the first 72 hr after treatment randomization from 1200 hr to 1200 hr next day, using collection bags attached to calves with medical glue. This technique is described in Figure 1 and is preferred over metabolic crates as it is less stressful for calves younger than 6 weeks of age. A daily representative sample of 400 g of faeces and two times 40 mL of urine were taken from the combined total collection material after thorough mixing. In addition, faecal spot samples were taken from day 2 to 5 by manual stimulation of rectum. The aetiology of the diarrhoea was not determined. All samples were transported in boxes with cooling elements and stored at −18°C.

### 2.4 Chemical analyses

Feed, urine, faeces and blood samples were processed and analysed at MasterLab (Boxmeer, the Netherlands). Samples of MR and treatments were analysed for dry matter (DM), crude ash, crude fat, crude protein, macro‐minerals, carbohydrates (lactose and glucose) and propionate. Faecal samples were analysed for DM, pH and macro‐minerals. Urine samples were analysed for macro‐minerals, urea, creatinine, pH and osmolality. Serum samples were analysed for macro‐minerals, urea and creatinine, while plasma samples were analysed for glucose. Dry matter content was determined by drying to a constant weight in a 103°C stove for 4 hr (EC 152/2009; EC, 2009). Crude ash was analysed by incineration in a muffle furnace by combustion for 4 hr at 550°C (EC 152/2009; EC, 2009). Crude fat was determined by treating the sample with hydrochloric acid and subsequent extraction with petroleum (EC 152/2009; EC, 2009). Crude protein content was analysed by combustion, according to the Dumas method (Etheridge, Pesti, & Foster, 1998; ISO 16634‐1:^2008^). Macro‐minerals were analysed using inductively coupled plasma mass spectrometry (PerkinElmer ICP‐MS 300D) according to NEN‐EN 2017 (2017). Chloride was analysed as described in Wilms et al. (2019). Carbohydrates in MR and treatments were determined by titrimetric method according to 1971 71/250/EEG for lactose and EG 152/2009 for glucose. The determination of propionic acid in solution was performed according to Canale, Valente, and Ciotti (1984). Separation of the organic acids was performed using the HPLC exclusion chromatography with UV and/or RI detection. Faecal and urine pH were measured using a calibrated pH meter according to NEN‐EN‐ISO 10523. Osmolality of urine was measured using a semi‐micro freezing point depression osmometer (K‐7400S, Knalier). Urea was analysed by two‐step enzymatic colorimetric analyses, hydrolysing urea to ammonium and CO_2_. Ammonium ions were analysed using a modified Berthelot reaction (10505, Human Diagnostics). Creatinine was analysed by a kinetic colorimetric analysis, based upon the Jaffe reaction (10051, Human diagnostics). Glucose was analysed by a colorimetric assay using a glucose oxidase–peroxide reaction. The colour intensity of the end product of this reaction (H_2_O_2_) was measured by absorbance methods (Enzychrom Glucose Assay kit, BioAssay Systems). In addition, serum total protein, serum albumin and globulin were analysed at the Animal Health Service (Gezondheidsdienst voor Dieren, Deventer, the Netherlands) using a Synchron Clinical Analyzer (Unicel DxC 800 SN4764, Beckman Coulter, Canada L.P.). Finally, plasma d‐Lactate was analysed at Hannover University and was determined enzymatically (combined d‐LDH and dGPT method).

### 2.5 Calculations and statistical analysis

The power was chosen to be equal to 80%. One of the most relevant parameters to consider in calf diarrhoea is blood pH. Based on the outcome of a previous experiment conducted at the Calf Research Facility of Trouw Nutrition Research and Development (Sint Anthonis, the Netherlands; unpublished data) investigating ORS efficacy, for 15 calves per treatment group (including an ORS group and a control group) on day 4 of diarrhoea, a standard deviation of 0.054 was assumed for blood pH. The minimal meaningful difference was considered to be 0.05. The minimal sample size to detect differences would then be 18 calves per treatment group.

Osmolality was calculated according to Stockham and Scott (2008) as followed:

$${\text{Osmolarity}}_{\text{blood}}\left(\text{mOsm}/L\right)=2\times \left({\left[{\text{Na}}^{+}\right]}_{\text{blood}}+{\left[K^{+}\right]}_{\text{blood}}\right)+{\left[\text{Glucose}\right]}_{\text{blood}}+{\left[\text{Urea}\right]}_{\text{blood}}$$

The SID was calculated according to Cave and Koo (2015) as followed:

$$\begin{array}{c} {\left[\text{SID}\right]}_{\text{blood}}\left(\text{mEq/L}\right)={\left[{\text{Na}}^{+}\right]}_{\text{blood}}+{\left[K^{+}\right]}_{\text{blood}}-({\left[{\text{Cl}}^{-}\right]}_{\text{blood}}\\ +{\left[{D\;-\;\text{lactate}}^{-}\right]}_{\text{blood}}+{\left[{L\;-\;\text{lactate}}^{-}\right]}_{\text{blood}}) \end{array}$$

The anion gap (AG) was calculated according to Trefz, Lorch, Feist, Sauter‐Louis, and Lorenz (2012) as followed:

$${\left[\text{AG}\right]}_{\text{blood}}\left(\text{mEq}/L\right)={\left[{\text{Na}}^{+}\right]}_{\text{blood}}={\left[K^{+}\right]}_{\text{blood}}-\left({\left[{\text{Cl}}^{-}\right]}_{\text{blood}}+\left[{\text{HCO}}_{3}^{-}\right]\right)$$

The strong ion gap (SIG) was calculated according to Constable (2014) as followed:

$${\left[\text{SIG}\right]}_{\text{blood}}\left(\text{mEq}/L\right)={\left[\text{total protein}\right]}_{\text{blood}}\times (0{\text{.343/\textbackslash{}\{ 1 + 10}}^{7\text{.08 - pH}}\text{\textbackslash{}\} )}-{\left[\text{AG}\right]}_{\text{blood}}$$

Urine SID was calculated as followed:

$${\left[\text{SID}\right]}_{\text{urine}}\left(\text{mEq}/L\right)={\left[{\text{Na}}^{+}\right]}_{\text{urine}}+{\left[K^{+}\right]}_{\text{urine}}-{\left[{\text{Cl}}^{-}\right]}_{\text{urine}}$$

The calculated change in plasma volume (PV) throughout the five monitoring was assessed from the serum total protein concentration measured on day 1 (*P_t_*_=0_) prior to ORS administration and the serum total protein concentration in the following days (*P_t_*_=_ *_x_*) according to van Beaumont, Greenleaf, and Juhos (1972):

$$\%\Delta \text{PV}=\left(P_{t=0}-P_{t=x}\right)\times 100/P_{t=x}$$

The SID intake was calculated using MR and treatment intakes. The SID of the solution (MR or treatments) was multiplied with the corresponding ingested volume, and this was divided by BW measured in the morning of day 1 (prior to treatment administration). The SID intakes was first calculated by only including treatment intakes and then by including treatment and MR intakes.

Similarly, BW measured in the morning of day 1 was used to express intakes, as well as faecal and urinary water and mineral losses per unit of BW. Water and mineral balances measured in the 24 hr after treatment randomization (day 1) were calculated as the difference between intakes (MR, treatment and supplemental water) and faecal and urinary losses.

Continuous variables were analysed using mixed‐model analysis with PROC MIXED in SAS (SAS 9.4M6, SAS^®^ Studio, SAS Institute). The statistical model was as follows:

$$Y_{\mathrm{ijkl}}=\mu_{0}+\mu +T_{i}+V_{j}+W_{k}+TW_{\mathrm{ik}}+C_{l}+e_{\mathrm{ijkl}}$$

where *Y_ijkl_* is the dependent variable, *µ* is the overall mean, *T_i_* is the fixed effect of treatment, *V_j_* is the fixed effect of block, *W_k_* is the fixed effect of day used as a repeated measure, *TW_ik_* is the effect of treatment‐by‐day interaction, *C_l_* is the random effect of calf, and *e_ijkl_* is the residual. Blood parameters measured on day 1, prior to treatment administration, were used in the model as a baseline covariate (*µ*_0_). Similarly, BW measured on day 1 was used as a baseline covariate for the analysis of BW on day 5. The covariance structure with the minimum values of Akaike's information criterion was the heterogeneous autoregressive covariance and was used for all variables. A logarithmic transformation was performed when a parameter did not follow a normal distribution. Results are presented as least squares means (LSM) with the standard error of the means (*SEM*). The correlation between faecal water and faecal Na^+^ losses was analysed using the PROC CORR in SAS. Variables were considered significant at differences *p* ≤ .05.

## 3 RESULTS

### 3.1 General health and intakes

Parameters describing calves on day 0 and before treatment initiation did not differ between treatment groups (Table 2). Five calves that did not develop diarrhoea after arrival at the facility were removed post‐inclusion from the study, and data collected from these calves were not used in the statistical models. In addition, five calves (two in HYPER, two in CON and one in HYPO) required intravenous fluid infusions due to severe dehydration and metabolic acidosis and two calves (two in HYPER) required other health interventions. These animals were removed from the dataset on the day the intervention took place (one calf on day 1, five calves on day 3 and one calf on day 5) and data collected prior to the day of removal were used. This led to having 16 calves in each ORS group and 18 calves in CON. Milk replacer intakes over the first three days (4.3 kg/day; *p* = .38) and the last two days (4.7 kg/day; *p* = .57) of the monitoring period did not differ across treatments. Treatment intakes and SID intakes were higher for calves receiving ORS (2.7 kg/day and 3.7 mEq/kg BW/day, respectively) than for CON calves (1.3 kg/day and 0.0 mEq/kg BW/day; *p* < .001; Table 3). Total SID intake over the first three days including MR and treatment intakes was higher in calves receiving ORS (7.4 mEq/kg BW/day) than CON calves (3.8 mEq/kg BW/day; *p* < .001). On day 5, BW of calves receiving low tonicity ORS (45.9 kg) was higher than CON calves (44.6 kg), and BW in HYPO calves (46.0 kg) was higher than in HYPER calves (44.7 kg; *p* = .04).

**TABLE 2:** Values of parameters describing calves before and after treatment initiation (*n* = 66)

Item	Treatment^1^								*p*‐value
	CON		HYPO		ISO		HYPER	Treatment
	Mean	*SEM*	Mean	*SEM*	Mean	*SEM*	Mean		*SEM*
Before treatment initiation									
Age (day)^2^	21.3	1.07	20.5	1.18	24.4	1.24	22.1	1.12	.16
Initial body weight (kg)	43.9	1.07	44.3	1.18	46.0	1.18	43.5	1.13	.44
Rectal temperature (°C)	38.7	0.13	38.8	0.14	38.6	0.14	38.6	0.14	.85
Blood parameters^3^									
Sodium (mM)	134.5	1.80	134.2	1.99	134.8	1.98	138.5	1.89	.33
Glucose (mM)	4.47	0.32	4.32	0.35	4.26	0.35	4.09	0.34	.88
pH	7.37	0.019	7.34	0.020	7.34	0.021	7.37	0.019	.60
Base excess (mM)	−1.73	1.07	−3.79	1.18	−1.58	1.17	−0.20	1.12	.19
After treatment initiation									
End BW on day 5 (kg)^4^	44.6^a^	0.40	46.0^b^	0.43	45.8^bc^	0.40	44.7^ac^	0.48	.04

^a,b,c^Means with a different superscript are significantly different (*p* ≤ .05).

^1^ Treatments included three ORS: low Na^+^, low lactose (HYPO, *n* = 16), low Na^+^, low glucose (ISO, *n* = 16), high Na^+^, high glucose (HYPER, *n* = 16), and one control solution consisting of 5 g/L of whey powder (CON, *n* = 18).

^2^ Expressed as log *SEM*.

^3^ Blood parameters were measured at the location of origin in the morning of day 0 using a blood gas analyser.

^4^ BW was measured between 1100 and 1200 hr on day 1 (prior to treatment administration) and day 5. BW on day 1 was used as baseline covariate. End BW measured on day 5 included 16 calves in CON, 15 calves in HYPO, 16 calves in ISO and 13 calves in HYPER. This is because in addition to the five calves that did not develop diarrhoea, seven calves were removed from the study prior to day 5, five due to severe dehydration and metabolic acidosis as a consequence of diarrhoea, and two due to other health issues requiring a medical intervention.

**TABLE 3:** Daily intakes of milk replacer, treatments and supplemental water from day 1 to 3 in calves with naturally occurring diarrhoea (*n* = 66). From day 1 to 3, calves were fed milk replacer (2.5 L) twice daily at 0700 and 1630 hr and were then offered treatments (2.0 L) twice daily at 1300 and 2100 hr

Intakes^1^	Treatments^2^								*p*‐values		
	CON		HYPO		ISO		HYPER		Treat	Time	Treat × Time
	Mean	*SEM*	Mean	*SEM*	Mean	*SEM*	Mean	*SEM*			
Milk replacer	4.31	0.17	4.51	0.20	4.47	0.20	4.07	0.20	.38	.14	.87
Treatment	1.29^a^	0.22	2.79^b^	0.25	2.59^b^	0.24	2.59^b^	0.23	<.001	.01	.98
Supplemental water	1.22	0.16	1.08	0.17	1.21	0.17	0.96	0.17	.66	.33	.50
Total daily fluid intake^3^	8.54	0.050	9.91	0.054	9.84	0.053	9.05	0.055	.15	.008	.99
SID intake (mEq/kg BW/day)^4^											
From treatments	0.02^a^	0.41	4.14^b^	0.44	3.37^b^	0.42	3.47^b^	0.42	<.001	.02	.12
From MR and treatments	3.78^a^	0.47	8.14^b^	0.52	7.13^b^	0.50	6.91^b^	0.53	<.001	.006	.40

Note

Besides, supplemental water was available ad libitum.

^a,b^Means with a different superscript are significantly different (*p* ≤ .05).

^1^ Expressed in kg/day unless specified otherwise.

^2^ Treatments included three ORS: low Na^+^, low lactose (HYPO, *n* = 16), low Na^+^, low glucose (ISO, *n* = 16), high Na^+^, high glucose (HYPER, *n* = 16), and one control solution consisting of 5 g/L of whey powder (CON, *n* = 18).

^3^ Expressed as log *SEM*.

^4^ Strong ion difference (SID) intake was calculated using milk replacer (SID = 39 mEq/L) and treatment (SID = 74 mEq/L for ORS and 3 mEq/L for CON) intakes. The SID of the solution (MR or treatment) was multiplied with the corresponding ingested volume, and this was divided by BW measured on day 1 after arrival.

### 3.2 Acid–base balance

**FIGURE 2:**
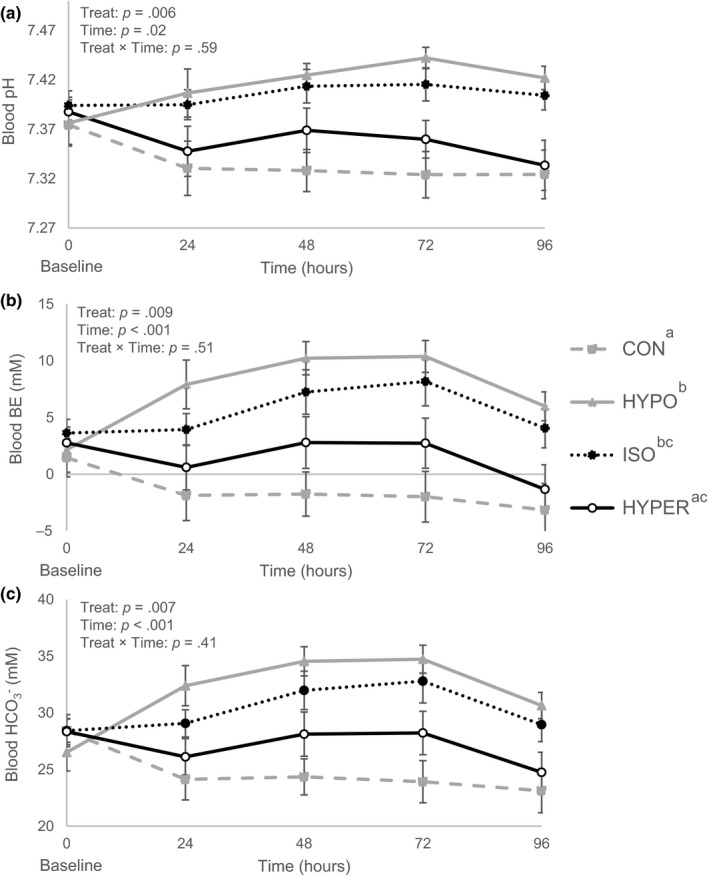
Blood acid–base balance (least square mean ± standard error) including pH (a), base excess (b) and HCO_3_ (c) measured daily at 1100 hr from day 1 to 5, in diarrhoeic calves receiving oral rehydration solutions (ORS) with different sodium chloride and sugar concentrations or a control solution. Treatments included three ORS: low Na^+^, low lactose (HYPO, *n* = 16), low Na^+^, low glucose (ISO, *n* = 16), high Na^+^, high glucose (HYPER, *n* = 16), and one control solution (CON, *n* = 18). From day 1 to 3, calves were fed MR (2.5 L) twice daily at 0700 and 1630 hr and were then offered treatments (2.0 L) twice daily at 1300 and 2100 hr. Significant treatment effects are indicated by different superscripts ^a,b,c^ (*p* ≤ .05). Standard errors were computed on raw data to better illustrate the observed differences in variability between treatments

Blood pH, BE, ${\text{HCO}}_{3}^{-}$ and tCO_2_ were higher in ISO and HYPO calves than in CON calves (*p* < .05; Table 4 and Figure 2), while HYPER did not differ with CON. In addition, blood pH, BE, ${\text{HCO}}_{3}^{-}$ and tCO_2_ were higher in HYPO than HYPER calves (*p* < .05), while ISO and HYPER tended to be different (*p* < .10). Serum SID was lower in CON calves (39 mEq/L) compared to calves receiving ORS (44 mEq/L; *p* = .03). Similarly, urine SID was lower in CON calves (8 mEq/L) compared to calves fed ORS (31 mEq/L; *p* = .001; Table 5). Serum SIG was lower in HYPER calves (2.8 mEq/L) compared to other groups (5.2 mEq/L; *p* = .02). There was a treatment effect on l‐Lactate being higher in HYPER (0.61 mM) than CON (0.46; *p* = .03). Blood pCO_2_ was lower in CON calves (44 kPa) compared to calves receiving ORS (48 kPa; *p* = .007).

**TABLE 4:** The effect of tonicity (as driven by NaCl, glucose and lactose) of oral rehydration solutions ( ORS) on blood minerals, blood haematology, and blood chemistry, blood acid–base balance, and blood gases measured daily at 1100 hr from day 1 to 5, in calves fed milk replacer and with naturally occurring diarrhoea (*n* = 66)

Item^1^	Treatments^2^								*p*‐values		
	CON		HYPO		ISO		HYPER		Treat	Time	Treat × Time
	Mean	*SEM*	Mean	*SEM*	Mean	*SEM*	Mean	*SEM*			
Blood minerals											
Sodium	128.9^a^	1.05	133.1^b^	1.00	132.3^b^	0.98	132.8^b^	1.09	.02	.76	.74
Potassium	5.29	0.09	5.52	0.10	5.40	0.09	5.41	0.10	.41	.002	.98
Chloride	97.9	1.19	96.7	1.21	95.4	1.18	95.4	1.36	.41	.61	.09
Calcium	2.69	0.02	2.76	0.03	2.74	0.03	2.72	0.03	.20	<.001	.82
Blood haematology											
Haematocrit (%)	31.5	0.72	31.1	0.75	30.2	0.74	32.3	0.76	.27	.18	.19
Blood chemistry											
Urea (mg/dL)^3^	24.0^a^	0.071	18.1^b^	0.074	17.3^b^	0.074	18.4^b^	0.081	.01	<.001	.32
Creatinine (mg/dL)^3^	1.08	0.027	1.02	0.028	1.02	0.023	1.06	0.061	.39	<.001	.55
Total serum protein (g/L)	55.1	0.53	53.8	0.57	53.9	0.54	55.1	0.58	.18	.34	.12
Albumin (g/L)	30.7	0.33	30.3	0.35	29.7	0.34	30.4	0.36	.23	.29	.30
Albumin to globulin ratio	1.32	0.01	1.32	0.01	1.30	0.01	1.30	0.01	.20	.01	.85
Glucose	5.00	0.184	5.50	0.199	5.31	0.185	5.16	0.201	.32	.32	.59
Serum osmolarity (mOsm/L)	283.7^a^	1.90	291.5^b^	2.03	287.7^ab^	1.89	290.0^b^	2.04	.04	.93	.87
Blood acid–base balance											
pH	7.33^a^	0.020	7.42^b^	0.021	7.41^bc^	0.021	7.35^ac^	0.022	.006	.02	.59
BE	−2.19^a^	2.22	8.63^b^	2.44	5.86^bc^	2.34	1.20^ac^	2.37	.009	<.001	.51
${\text{HCO}}_{3}^{-}$	23.9^a^	1.83	33.1^b^	2.03	30.7^bc^	1.94	26.8^ac^	1.96	.007	<.001	.41
SID (mEq/L)	38.6^a^	1.54	44.6^b^	1.64	43.8^b^	1.56	44.1^b^	1.69	.03	.51	.80
AG (mEq/L)	11.5	1.14	10.6	1.18	9.0	1.22	13.0	1.31	.18	.007	.99
SIG (mEq/L)^3^	4.75^a^	0.18	6.34^a^	0.17	4.53^a^	0.15	2.75^b^	0.19	.02	.06	.08
l‐Lactate	0.46^a^	0.059	0.54^ab^	0.063	0.54^ab^	0.062	0.61^b^	0.065	.03	.14	.84
d‐Lactate	0.33	0.21	0.12	0.22	0.47	0.21	0.33	0.22	.73	.08	.70
Blood gases											
tCO_2_	25.8^a^	2.00	34.6^b^	2.22	31.9^bc^	2.13	28.1^ac^	2.15	.03	<.001	.57
pCO_2_ (kPa)	43.8^a^	1.05	48.3^b^	1.13	48.9^b^	1.10	47.4^b^	1.14	.007	<.001	.60

Abbreviations: AG, anion gap; BE, base excess; SID, strong ion difference; SIG, strong ion gap.

^a,b,c^Means with a different superscript are significantly different (*p* ≤ .05).

^1^ Expressed in mM unless specified otherwise.

^2^ Treatments included three ORS: low Na^+^, low lactose (HYPO, *n* = 16), low Na^+^, low glucose (ISO, *n* = 16), high Na^+^, high glucose (HYPER, *n* = 16), and one control solution consisting of 5 g/L of whey powder (CON, *n* = 18).

^3^ Expressed as log *SEM*.

**TABLE 5:** The effect of tonicity (as driven by NaCl, glucose and lactose) of oral rehydration solutions (ORS) on urine acid–base balance, urine chemistry and faecal parameters measured in calves fed milk replacer and with naturally occurring diarrhoea (*n* = 66)

Item^1^, ^2^	Treatments^3^								*p*‐values		
	CON		HYPO		ISO		HYPER		Treat	Time	Treat × Time
	Mean	*SEM*	Mean	*SEM*	Mean	*SEM*	Mean	*SEM*			
Urine chemistry											
Urea^4^	279^a^	0.10	188^b^	0.10	212^b^	0.10	189^b^	0.11	.03	<0.001	0.10
Creatinine	21.8	1.25	21.4	1.20	21.2	1.14	19.4	1.28	.56	0.01	0.16
Urine osmolality (mOsm/kg)	395^a^	39	389^a^	41	375^a^	39	550^b^	40	.009	0.03	0.46
Urine SID (mEq/L)	7.9^a^	4.44	31.0^b^	4.88	30.9^b^	4.42	29.6^b^	4.73	.001	0.008	0.10
Urine pH^4^	8.50	0.015	8.22	0.015	8.29	0.015	8.43	0.015	.37	0.39	0.13
Faecal DM (%)^4^	10.7^a^	0.059	10.4^a^	0.062	10.9^a^	0.061	8.4^b^	0.065	.02	0.009	0.93
Faecal pH^4^	5.67	0.018	5.88	0.19	5.80	0.019	5.80	0.019	.60	0.008	0.98

^a,b^Means with a different superscript are significantly different (*p* ≤ .05).

Abbreviations: DM, dry matter; SID, strong ion difference.

^1^ Expressed in mg/kg BW/day, unless specified otherwise.

^2^ Urine parameters were measured by daily quantitative collection of urine in the first three days after treatment randomization (day 1 to 3). Faecal DM and pH were measured daily by total collection in the 24 hr after treatment randomization (day 1) and by spot sampling from day 2 to 5.

^3^ Treatments included three ORS: low Na^+^, low lactose (HYPO, *n* = 16), low Na^+^, low glucose (ISO, *n* = 16), high Na^+^, high glucose (HYPER, *n* = 16), and one control solution consisting of 5 g/L of whey powder (CON, *n* = 18).

^4^ Expressed as log *SEM*.

### 3.3 Blood electrolytes and chemistry

**FIGURE 3:**
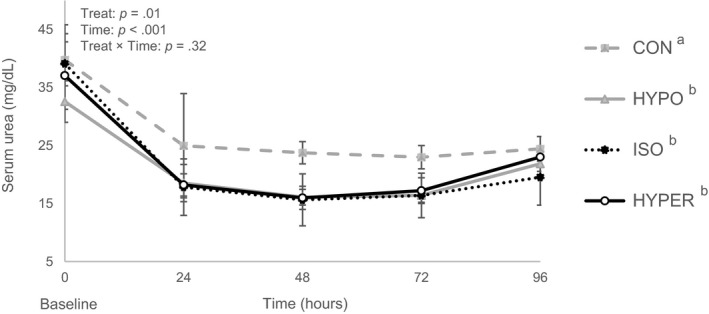
Serum urea (least square mean ± standard error) in diarrhoeic calves receiving oral rehydration solutions (ORS) with different sodium chloride and sugar concentrations or a control solution. Treatments included three ORS: low Na^+^, low lactose (HYPO, *n* = 16), low Na^+^, low glucose (ISO, *n* = 16), high Na^+^, high glucose (HYPER, *n* = 16), and one control solution (CON, *n* = 18). From day 1 to 3, calves were fed MR (2.5 L) twice daily at 0700 and 1630 hr and were then offered treatments (2.0 L) twice daily at 1300 and 2100 hr. Significant treatment effects are indicated by different superscripts ^a,b^ (*p* ≤ .05). Standard errors were computed on raw data to better illustrate the observed differences in variability between treatments

**FIGURE 4:**
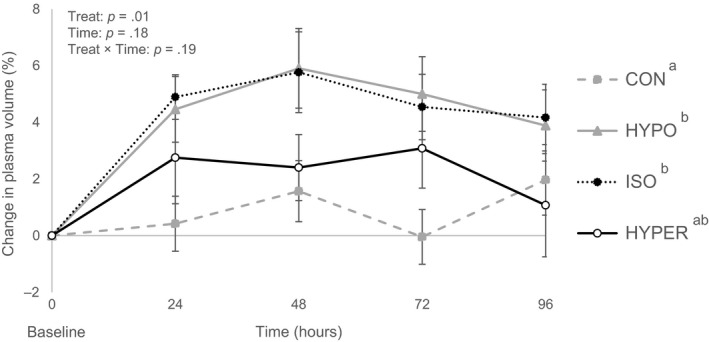
Calculated change in plasma volume (least square mean ± standard error) in diarrhoeic calves receiving oral rehydration solutions (ORS) with different tonicity (as driven by sodium chloride, dextrose or lactose) or a control solution. Treatments included three ORS: low Na^+^, low lactose (HYPO, *n* = 16), low Na^+^, low glucose (ISO, *n* = 16), high Na^+^, high glucose (HYPER, *n* = 16), and one control solution (CON, *n* = 18). From day 1 to 3, calves were fed MR (2.5 L) twice daily at 0700 and 1630 hr and were then offered treatments (2.0 L) twice daily at 1300 and 2100 hr. Significant treatment effects are indicated by different superscripts ^a,b^ (*p* ≤ .05). Standard errors were computed on raw data to better illustrate the observed differences in variability between treatments

Serum Na^+^ was constant in calves fed ORS (133 mM), while it was lower in CON calves (129 mM; *p* = .02). Serum urea was higher in CON calves (24 mg/dL) compared to ORS groups (18 mg/dL; *p* = .01; Figure 3). Similarly, urine urea was higher in CON calves (279 mg/kg BW/day) than calves fed ORS (196 mg/kg BW/day; *p* = .03). Blood glucose did not differ across treatment groups (5.2 mM; *p* = .32). Serum osmolarity was higher in HYPO and HYPER calves (291 mOsm/L) compared to CON calves (284 mOsm/L; *p* = .04), while ISO did not differ from other groups. In contrast, urine osmolality was higher for HYPER (550 mOsm/kg) than other treatment groups (386 mOsm/kg; *p* = .009). Serum total protein (54 g/L) did not differ amongst groups (*p* = .18). However, changes in plasma volume calculated using total protein concentrations were higher in HYPO and ISO (+4.8%) when compared with CON (+1.0%; *p* = .01; Figure 4). Additionally, HYPER (+2.8%) tended to be different from HYPO and ISO (*p* = .08).

### 3.4 Water and mineral balance

**FIGURE 5:**
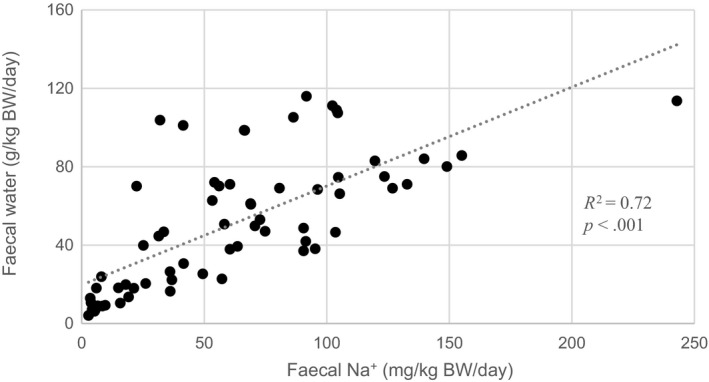
Correlation between faecal water and faecal Na^+^ losses measured in the 24 h after treatment randomization and administration in diarrhoeic calves fed oral rehydration solutions with different tonicity (as driven by sodium chloride, dextrose and lactose) or a control solution (*n* = 66)

Urine water losses were higher in HYPO and ISO calves (63 g/kg BW/day) than in HYPER calves (42 g/kg BW/day; *p* = .05; Table 6).In addition, urine water losses tended to be lower in HYPO and ISO than CON (46 g/kg/BW/day; *p* < 0.10). Faecal water losses tended to be lower in HYPO calves (26 g/kg BW/day) compared to CON and HYPER calves (43 g/kg BW/day; *p* = .08). Sodium intake was the highest in HYPER calves (231 mg/kg BW/day) and the lowest in CON calves (57 mg/kg BW/day; *p* < .001). Urine Na^+^ losses were higher in calves fed ORS (36 mg/kg BW/day) when compared with CON calves (4 mg/kg BW/day; *p* = .003). Faecal Na^+^ losses were lower in calves receiving low tonicity ORS (45 mg/kg BW/day) compared with HYPER calves (76 mg/kg BW/day; *p* = .04). There was a strong correlation between faecal Na^+^ and faecal water losses (*R* ^2^ = 0.72; *p* < .001; Figure 5). Faecal DM was lower in HYPER (8%) compared to other groups (11%; *p* = .02) throughout the five monitoring days. Sodium balance was higher in ORS calves (110 mg/kg BW/day), while it was only 3 mg/kg BW/day in CON calves (*p* < .001). Potassium intake (241 mg/kg BW/day) and urine K^+^ losses (97 mg/kg BW/day) were higher in calves receiving ORS when compared to CON calves (178 and 61 mg/kg BW/day, respectively; *p* < .05). Potassium balance tended to be higher in HYPO (102 mg/kg BW/day) than CON calves (39 mg/kg BW/day; *p* = .08). Chloride intake was the highest in HYPER calves (300 mg/kg BW/day) and the lowest in CON calves (138 mg/kg BW/day; *p* < .001). Urine Cl^−^ losses were higher in HYPER calves (115 mg/kg BW/day) compared to CON calves (59 mg/kg BW/day; *p* = .05). The Cl^−^ balance was higher in calves fed HYPO and HYPER (121 mg/kg BW/day), than in CON calves (37 mg/kg BW/day; *p* = .04). Faecal Ca^2+^ losses were greater in CON (100 mg/kg BW/day) than HYPER (67 mg/kg BW day; *p* = .05).

**TABLE 6:** The effect of tonicity (as driven by NaCl, glucose and lactose) of oral rehydration solutions (ORS) on water and mineral balance measured in the first 24 hr after treatment randomization in calves fed milk replacer and with naturally occurring diarrhoea (*n* = 66)

Item^1^, ^2^	Treatments^3^								*p*‐values
	CON		HYPO		ISO		HYPER		Treat
	Mean	*SEM*	Mean	*SEM*	Mean	*SEM*	Mean	*SEM*	
Water balance (g/kg BW/day)									
Intake	149.3	12.7	182.8	13.7	176.8	13.7	158.5	13.7	.25
Urine losses	45.8^ab^	6.6	62.2^a^	7.0	64.6^a^	6.6	42.3^b^	7.0	.05
Faecal losses^4^	43.9	0.14	25.7	0.16	37.9	0.17	41.1	0.15	.08
Balance	53.3	10.6	89.2	11.2	70.2	11.1	62.7	11.2	.14
Sodium balance									
Intake	56.9^a^	16.0	187.4^b^	17.3	180.8^b^	17.3	230.6^c^	17.3	<.001
Urine losses	3.8^a^	7.4	28.6^b^	7.7	35.4^b^	7.3	44.8^b^	7.7	.003
Faecal losses	58.4^ab^	7.9	40.2^a^	9.4	49.8^a^	9.0	75.9^b^	8.3	.04
Balance	2.8^a^	19.1	119.6^b^	20.3	99.1^b^	20.2	111.5^b^	20.3	<.001
Potassium balance									
Intake	177.5^a^	16.9	259.4^b^	18.4	244.4^b^	18.3	218.3^ab^	18.4	.01
Urine losses	61.1^a^	9.6	100.3^b^	10.1	103.3^b^	9.6	88.8^b^	10.1	.02
Faecal losses^4^	66.6	0.17	49.0	0.18	51.4	0.19	50.5	0.18	.57
Balance	38.7	16.4	101.7	17.4	70.4	17.3	56.6	17.4	.08
Chloride balance									
Intake	137.6^a^	19.3	224.8^b^	20.9	216.3^b^	20.8	300.3^c^	20.9	<.001
Urine losses	58.5^a^	13.6	83.4^ab^	14.2	89.2^ab^	13.5	115.0^b^	13.7	.05
Faecal losses^4^	27.8	0.22	21.9	0.24	25.4	0.25	37.8	0.23	.42
Balance	36.6^a^	23.1	115.8^b^	23.3	95.7^ab^	23.0	125.6^b^	23.3	.04
Calcium balance									
Intake	110.7	8.3	119.0	9.0	111.7	9.3	100.5	9.0	.53
Urine losses^4^	0.94	0.34	0.52	0.32	0.73	0.32	1.10	0.34	.38
Faecal losses	100.1^a^	7.94	82.5^ab^	8.62	85.8^ab^	9.34	67.0^b^	8.29	.05
Balance	9.5	11.1	42.5	10.6	26.7	10.5	32.8	11.1	.19

^a,b,c^Means with a different superscript are significantly different (*p* ≤ .05).

^1^ Expressed in mg/kg BW/day unless specified otherwise.

^2^ Water and mineral balances calculated as the difference between intakes (milk replacer, treatment and supplemental water) and faecal and urine losses per unit of body weight.

^3^ Treatments included three ORS: low Na^+^, low lactose (HYPO, *n* = 16), low Na^+^, low glucose (ISO, *n* = 16), high Na^+^, high glucose (HYPER, *n* = 16), and one control solution consisting of 5 g/L of whey powder (CON, *n* = 18).

^4^ Expressed as log *SEM*.

## 4 DISCUSSION

The aim of the present study was to assess the effect of ORS tonicity (as driven by NaCl, glucose and lactose) on water, mineral, and acid–base balance in calves with naturally occurring diarrhoea. Daily intakes of the three experimental ORS in terms of volume and SID per unit of BW did not differ, thus allowing fair comparison amongst the three ORS treatments. Calves in the low tonicity groups (HYPO and ISO) had higher BW (+1 kg) at the end of the five monitoring days compared with other treatments. This was associated with a greater plasma volume expansion, a higher alkalinizing capacity, as well as lower faecal Na^+^ losses for low tonicity ORS when compared with the hypertonic treatment.

### 4.1 Acid–base balance

Oral rehydration solutions designed for diarrhoeic calves require a concentration of alkalinizing agents between 50 and 80 mM, and a SID of at least 60 mM to maintain or restore blood acid–base balance (Smith & Berchtold, 2014). This is because the strong ion theory states that ORS should deliver an excess of strong cations (Na^+^ and K^+^) relative to the concentration of strong anions (Cl^−^). Blood pH and BE of calves in the HYPO and ISO groups remained within the normal range for male calves (7.38–7.46 and 2.6–10.8 mM, respectively; Dillane, Krump, Kennedy, Sayers, & Sayers, 2018), whereas CON and HYPER calves did not. In addition, calves receiving low tonicity ORS had higher concentrations of ${\text{HCO}}_{3}^{-}$ and tCO_2_ compared with HYPER and CON calves. The lower blood pH in CON calves was likely the consequence of lower Na^+^ concentrations in blood resulting in lower blood and urine SID in this group. In HYPER calves, the lower blood pH levels may be linked with an increase of unmeasured strong anions as shown by the slightly lower blood SIG in HYPER calves (Constable, 2014). This was associated with higher l‐lactate concentrations in HYPER than CON but differences were of low magnitude.

Despite having the same SID and the same propionate concentration (from Na propionate), low tonicity ORS were superior to HYPER in maintaining and restoring blood acid–base balance. According to Lifshitz and Wapnir (1985), the optimal ORS Na^+^ concentration to maximize intestinal Na^+^ and water absorption in rats is between 60 and 80 mM. As faecal Na^+^ losses were approximately twofold higher in HYPER than in HYPO calves in the 24 hr after treatment randomization and administration, this suggests that part of the Na^+^ content of the HYPER treatment (134 mM) was not absorbed by the animals. Sodium absorption is almost exclusively carried out by transcellular absorption, which is a saturable process (Goff, 2018). Thus, there is a limited capacity to transport Na^+^ across the epithelial cells (Goff, 2018). In contrast, Cl^−^ is absorbed more efficiently by the gastrointestinal tract (Goff, 2018), which is supported by the similar faecal Cl^−^ losses between all three ORS treatments. This suggests that the Cl^−^ load from the HYPER treatment was absorbed to a greater extent than Na^+^, therefore lowering the effective SID of the HYPER treatment. However, the similar urine SID across ORS groups does not support this hypothesis. Finally, the use of lactose over dextrose did not affect the alkalinizing capacity as the HYPO and ISO treatments did not differ regarding blood pH, BE and ${\text{HCO}}_{3}^{-}$ . This suggests that a reduction of approximately 50 mOsm/kg in osmolality does not improve solution efficacy regarding maintenance of acid–base balance.

### 4.2 Water and mineral balance

Oral rehydration solutions for diarrhoeic animals usually contain high Na^+^ concentrations (>70 mM) to compensate for diarrhoeal faecal Na^+^ losses and, thus, to replenish the extracellular fluid volume (Smith & Berchtold, 2014). Serum Na^+^ remained constant in calves receiving ORS, while CON calves developed hyponatremia (serum Na^+^ <130 mM; Byers, Lear, & Van Metre, 2014). The lower serum Na^+^ in CON calves was the consequence of faecal Na^+^ losses exceeding the daily Na^+^ intake. Although the HYPER treatment was higher in Na^+^ (134 mM), none of the HYPER calves developed hypernatremia, which suggests that calves consumed an adequate volume of supplemental water (1 kg/day; Wilms, Leal, et al., 2020). Serum urea concentrations and urine urea content were lower in calves fed ORS, indicating that all three ORS were able to rehydrate animals to some extent, thus allowing for urea excretion through urine (Higgins, 2016). Control calves had lower urine water and Na^+^ losses, which may be attributed to an increased secretion of aldosterone and antidiuretic hormone resulting in water and Na^+^ retention to mitigate dehydration (Byers et al., 2014). The higher mineral excretion in urine, as well as the lower urine volume in HYPER calves, led to a 30% increase in urine osmolality when compared to other groups. In contrast, the higher urine volumes, as well as the lower urine osmolality in low tonicity groups, may indicate recovery from diarrhoea and improved hydration status (Perrier et al., 2015; Thornton & English, 1976). This was supported by the increase in plasma volume expansion, which was greater in HYPO and ISO calves (+4.8%), as compared with HYPER (+2.8%) and CON (+1.0%).

Normal faecal water losses in calves are 4.3 ± 1.0 g/kg BW/day (Lewis & Phillips, 1972). Faecal water losses from calves in the current study were 5 to 10‐fold higher, which is consistent with previous results from Lewis and Phillips (1972), where diarrhoeic calves had faecal water losses of 47.7 ± 3.4 g/kg BW/day. However, averages for faecal water losses include high variation, as calves in the present study had faecal water losses up to 178 g/kg BW/day, which is equivalent to 8 kg/day of faeces for a 45 kg calf. Calves in the HYPO treatment tended to have lower faecal water losses compared to CON and HYPER calves, suggesting that low tonicity ORS containing lactose may be more effective than hypertonic ORS at mitigating diarrhoeal water losses. This was associated with lower faecal DM throughout the five monitoring days in HYPER calves, suggesting long‐lasting intestinal disturbances in that group.

Faecal Na^+^ losses of calves involved in this experiment were lower (51 ± 7 mg/kg BW/day) than those reported by Lewis and Phillips (1972), where calves younger than 1 week of age with naturally occurring diarrhoea had faecal Na^+^ losses of 195.6 ± 73.3 mg/kg BW/day. The current study was performed on a larger scale (*n* = 66) and with older calves than the study from Lewis and Phillips (*n* = 3; 1971), and was likely more representative of the overall population of diarrhoeic calves between 15 and 29 day of age. In the current study, faecal Na^+^ losses were as high as 243 mg/kg BW/day in the most severe case, which still remains below those of diarrhoea from cholera origin, where faecal Na^+^ losses can exceed 500 mg/kg BW/day (Harris, LaRocque, Qadri, Ryan, & Calderwood, 2012; Table 7). Faecal Na^+^ losses of low tonicity groups were 41% lower than those in HYPER calves, while CON did not significantly differ from other treatment groups. This indicates that low tonicity ORS (≤300 mOsm/kg) with low Na^+^ concentrations (<90 mM) may allow for mitigation of diarrhoeal losses when compared with hypertonic ORS (>400 mOsm/kg). Despite lower total daily Na^+^ intakes in CON calves, faecal Na^+^ losses were numerically higher than those of HYPO and HYPER calves. As CON calves did not receive any ORS, their health status declined, which may have resulted in increased diarrhoeal losses. However, the lack of significance, as well as the absence of pathogen evaluation in faeces, does not allow to discuss this.

**TABLE 7:** Faecal Na^+^ losses over 24 hr in children and calves with diarrhoea from various aetiologies

Diarrhoea aetiology	Subject	Age	Sample size	Faecal Na^+^ losses^1^	References
Healthy	Calves	<1 week	*n* = 7	9.5 ± 2.5	Lewis and Phillips (1972)
Rotavirus	Children	11 ± 1 months	*n* = 28	57	Sack et al. (1978)
		17.1 ± 1.2 months	*n* = 45	75	Molla et al. (1981)
ETEC	Children	19.2 ± 13.1 months	*n* = 38	120.7	Molla et al. (1981)
Cholera	Children	–	–	up to 552	Harris et al. (2012)
		37.6 ± 16.0 months	*n* = 37	247	Molla et al. (1981)
Diarrhoea challenge model	Calves^2^	<1 week	*n* = 2	167.9 ± 70.9	Lewis and Phillips (1972)
	Calves^3^	<1 week	*n* = 3	319.7 ± 81.7	Lewis and Phillips (1972)
Naturally occurring diarrhoea, mixed pathogens	Calves	<1 week	*n* = 3	195.6 ± 73.3	Lewis and Phillips (1972)
	Calves	CON: 21.3 ± 1.1 day HYPO: 20.5 ± 1.2 day ISO: 24.4 ± 1.2 day HYPER: 22.1 ± 1.1 day	CON: *n* = 18 HYPO: *n* = 16 ISO: *n* = 16 HYPER: *n* = 16	CON: 58 (29.9/91.7) HYPO: 40 (13.4/74.6) ISO: 50 (26.9/65.1) HYPER: 76 (32.0/104.0)	Current study^4^, ^5^

^1^ Expressed in mg/kg BW/day.

^2^ Diarrhoea induced by feeding 2 g/kg BW of sucrose.

^3^ Diarrhoea induced by feeding intestinal contents and mucosal scrapings from calves that had died from naturally occurring diarrhoea.

^4^ Treatments included three oral rehydration solutions: low sodium, low lactose (HYPO, *n* = 16), low sodium, low glucose (ISO, *n* = 16), high sodium, high glucose (HYPER, *n* = 16), and one control solution consisting of 5 g/L of whey powder (CON, *n* = 18).

^5^ Values for faecal Na^+^ losses are given as medians and 25‐/75‐quartiles measured in the first 24 hr after treatment randomization (day 1).

According to Wapnir and Lifshitz (1985), glucose concentrations between 80 and 100 mM allow for optimal water and Na^+^ absorption. The higher faecal output (water and Na^+^) in HYPER calves may therefore be explained by a reduced water absorption resulting from the high amount of Na^+^ (134 mM) and glucose (151 mM) present in the solution. Non‐absorbed nutrients increase the osmolality of the gut content, thus increasing the secretion of water towards the lumen of the gut and exacerbating diarrhoea severity. This is illustrated by the strong correlation between faecal water and Na^+^ losses found in the current study. Besides the possible saturation of Na^+^ absorption pathways, the limited apparent Na^+^ absorption in the HYPER group may also be associated with a reduction of the intestinal absorptive surface as a direct consequence of diarrhoea (Klein et al., 2008). The immune status of calves, as well as the diarrhoea aetiology, may also have influenced the outcome of this experiment in relation to development of metabolic acidosis and diarrhoeal losses. However, adequate randomization and blocking of calves may have prevented having unbalanced groups in terms of diarrhoea severity.

Currently, the Centers for Disease Control and Prevention recommends low tonicity ORS (~300 mOsm/kg) for children with diarrhoea and dehydration, irrespectively of the cause (King et al., 2003). Results of the current experiment show that similar fluids could also be effective in rehydrating and mitigating metabolic acidosis severity in calves (Foster & Smith, 2009). Despite higher faecal Na^+^losses, lower fecal DM, and higher urine osmolality, the HYPER treatment showed similar efficacy than the low tonicity ORS regarding the maintenance of serum electrolytes, serum osmolarity and serum urea concentrations. However, the plasma volume expansion and the alkalinizing capacity of the HYPER treatment were lower than that of low tonicity ORS. This suggest that current conventions for Na^+^, and possibly for glucose concentrations, in ORS for diarrhoeic calves are too high. This study also demonstrated that the SID of an ORS is therefore not the only determinant when assessing the alkalinizing capacity of a product. Results of the current study are dependent on the tested ORS formulas, and further work is needed to evaluate the effect of ORS tonicity in relation to diarrhoea aetiology.

## 5 CONCLUSION

Results of this experiment show that despite being formulated to have a similar alkalinizing capacity, oral rehydration solutions with lower sodium concentration, and thus lower tonicity, are superior at restoring and maintaining acid–base balance compared to a hypertonic solution when offered to calves fed milk replacer and with naturally occurring diarrhoea. This suggests that oral rehydration with a high mineral content, particularly with respect to sodium and chloride, may impair the alkalinizing capacity of the solution and increase faecal output and urine osmolality. These data therefore indicates that low tonicity oral rehydration solutions may be more effective at restoring water, mineral and acid–base balance in diarrhoeic calves. Whether the observed differences are clinically relevant requires further work.

## CONFLICT OF INTEREST

The present study was funded by Trouw Nutrition (Amersfoort, the Netherlands), a company with commercial interests in oral rehydration solutions.

## ETHICAL APPROVAL

The authors confirm that the ethical policies of the journal, as noted on the author guidelines page, have been adhered to, and the appropriate ethical review committee approval has been received. All procedures described in this article complied with the Dutch Law on Experimental Animals, which complies with ETS123 (Council of Europe 1985 and the 86/609/EEC Directive), and were approved by the Animal Welfare Authority (DEC Utrecht, the Netherlands).
